# The prevalence and pattern of antibiotic prescription among insured patients in Dar es Salaam Tanzania

**DOI:** 10.11604/pamj.2021.40.140.29584

**Published:** 2021-11-05

**Authors:** Mohamed Ally Khalfan, Philip Galula Sasi, Sabina Ferdinand Mugusi

**Affiliations:** 1 Department of Clinical Pharmacology, School of Medicine, Muhimbili University of Health and Allied Health Sciences, Dar es Salaam, Tanzania

**Keywords:** Antibiotic prescription prevalence, antibiotic prescription pattern, 2019 WHO AWaRe classification

## Abstract

**Introduction:**

high prevalence of antibiotic prescriptions may contribute to the problem of antibiotic resistance. Understanding the pattern of antibiotic prescriptions in a country may inform monitoring and stewardship activities, which are crucial in the fight against antibiotic resistance. We aimed to determine the prevalence and describe the pattern of antibiotic prescriptions among National Health Insurance Fund (NHIF) insured patients receiving treatment at health facilities in Ilala Municipality, Dar es Salaam, Tanzania.

**Methods:**

a cross-sectional analysis of claim forms of NHIF insured patients. A data extraction form was used to capture data for September, 2019 submitted to the Ilala NHIF offices.

**Results:**

among 993 insured patients (mean [±SD] age 36.3 [±23.2] years; 581 [58.5%] females; 535 [53.9%] adults) a total of 357 (46.4%, 95% CI, 42.8-50.0) received an antibiotic prescription. Of the 357 patients who received an antibiotic prescription, 71(19.9%) received more than one antibiotic prescription. The most common antibiotic prescribed was amoxicillin/clavulanate (17.1%) followed by amoxicillin (16.5%) whereas the most commonly prescribed antibiotic class was the penicillins (51.3%) followed by the nitroimidazoles (14.0%). Among patients who received more than one antibiotic, the most commonly co-prescribed antibiotics were Ampicillin/Cloxacillin plus Metronidazole (11.4%) followed by Amoxicillin plus Metronidazole (7.1%). According to 2019 WHO Access, Watch, Reserve (AWaRe) Classification of antibiotics, 60.8% of patients received the access antibiotics, 33.3% received the watch antibiotics whereas 17.4% of patients received antibiotics that were not recommended. No patient received an antibiotic from the reserve group.

**Conclusion:**

the prevalence of antibiotic prescriptions in Tanzania is high and some antibiotics not recommended by the WHO are still prescribed. We recommend revision of the current Tanzania treatment guideline on antibiotics to reflect WHO recommendations, and further research to address local factors influencing antibiotic prescriptions is warranted.

## Introduction

Discovery of antibiotics has revolutionized medicine in terms of clinical outcomes of various infectious diseases which prior to that were untreatable and deadly [1,2]. Moreover, antibiotics have changed the way we approach major surgery, organ transplantation, cancer patients, and chronic immunodeficiency syndromes both therapeutically and prophylactically [1,3]. However, overuse and unregulated use of existing antibiotics, especially those of last resort, has the potential to compromise their efficacy largely due to the boosting and dissemination of defiant pathogens [1]. To date, the discovery of new antibiotics remains a rare phenomenon and this in part exacerbates the development and spread of antibiotic resistance [1,3,4]. There is a global deficit of new classes of antibiotics that help fight multidrug-resistant bacteria which can account for relentless infections with a mortal sequel [3,5].

Antibiotic prescription rate from healthcare settings is increasing in many countries and in fact, sometimes antibiotics are over prescribed [6,7]. Despite the conditions like colds and flu being viral infections and cannot be treated by antibiotics, yet patients receive a prescription for such problems [8]. Antibiotic prescription rate constitutes one of the core prescribing indicators for determining the Index of Rational Drug Prescribing (IRDP) for a given health facility and it has been argued that, the optimal level is being less than 30% [9]. The antibiotic prescription epidemic is also relevant in Tanzania as shown by a study by Irunde and colleagues in four regions in the country [7]. The authors showed that antibiotic prescribing in Tanzania is far from being rational. Antibiotic prescribing together with other core prescribing indicators in Tanzania were sub-optimal of which an average antibiotic prescription prevalence of 67.7% was found. The problem of increased antibiotic prescriptions has been, in part, exacerbated by the introduction of a rapid diagnostic test for malaria (MRDT). This is because any febrile patient found to be negative for malaria using MRDT is now more likely to receive an antibiotic prescription than before [10,11]. In an effort to equip countries with an interactive tool to support monitoring and stewardship activities, the World Health Organization (WHO) Expert Committee on Selection and Use of Essential Medicines recommended the development of the 2019 WHO AWaRe (Access, Watch, Reserve) Classification Database. This database classifies antibiotics into three stewardship groups: Access, Watch and Reserve, emphasizing the importance of their optimal uses and antimicrobial resistance potential. Moreover, the classification also includes a list of antibiotics not recommended by the WHO, which include fixed-dose combinations of multiple broad spectrum antibiotics lacking evidence-based indications [12].

The access group include antibiotics that are active against commonly encountered susceptible pathogens while also showing lower resistant potential than antibiotics in other groups. The World Health Organization (WHO) specifies a country-level target of at least 60% of antibiotics consumed should be from the Access group. Watch group include antibiotics classes that have higher resistance potential and includes most of the highest priority agents among the critically important antimicrobials for human medicine and/or antibiotics that are at relatively high risk of selection of bacterial resistance. The Reserve group, treated as ‘´last resort´´ option, include antibiotics that should be reserved for treatment of confirmed or suspected infections due to multi-drug-resistant organisms [12]. Studies from other countries have shown that being insured is associated with a high prevalence of antibiotic prescription when compared with being uninsured [13,14]. The NHIF is the main health insurance scheme in Tanzania and it has been running for about two decades with a current coverage of about 9% and the scheme is growing [15]. In view of expanding health insurance scheme in Tanzania the prevalence of antibiotic prescriptions may be higher than previously reported. In addition, there is limited data on antibiotic prescription prevalence and pattern among insured patients in Tanzania. We aimed to determine the prevalence and describe the pattern of antibiotic prescriptions among insured patients.

## Methods

**Study setting and population**: the study was conducted in 73 NHIF-accredited health-care facilities in Ilala Municipality in Dar es Salaam, Tanzania. Ilala is the most populous municipality in Dar es Salaam, the commercial hub of Tanzania. The population in Ilala municipality was projected to be about 1.6M people in 2017 [16]. The municipality has an area of 273 km^2^ [17]. Ilala municipality has the largest number of national referral hospitals and specialists in Tanzania, therefore it was purposefully selected. Since the inception of NHIF in 2001, there has been an increase in the number of accredited health facilities offering services to both public and private NHIF beneficiaries. The majority of NHIF-accredited health facilities offered primary care at dispensary level [15]. This study included all NHIF insured patients who received treatment from Ilala Municipality health facilities during the month of September 2019. Inclusion criteria was all accessible NHIF claim forms and claim forms that had been filled by a non-prescriber health worker such as physiotherapist or occupational therapist, and those with missing prescriber information were excluded.

**Study design, sample size and sampling procedures**: the study was a cross-sectional analysis of the NHIF claim forms that were filled from Ilala Municipality health-care facilities. The minimum sample size was estimated using the formula by Kirkwood and Sterne [18]. Assuming the antibiotic prescription prevalence, P is 67.7% from a previous study [7] and ε is the margin of error, 5% (0.05). Thus, the minimum sample size was calculated to be about 344 patients. Including a 10% of possible forms missing prescriber information, the minimum sample size for this study increased to 378. However, we decided to include a total of 1100 claim forms as our final sample size because we wanted to improve precision of our estimates to answer all specific objectives including to be able to perform sub-group analysis. There was no added risk to patients as a result of increasing the sample size and it was affordable as the data was readily available. The sampling frame was a list of all claim forms for the month of September 2019 from Ilala Municipality health facilities. The total number of claim forms in the sampling frame was 125,695. Simple random sampling using a table of random numbers from the Open Source Epidemiologic Statistics for Public health (OpenEpi) website was used to obtain our target sample size of 1100 claim forms from the sampling frame.

**Data collection methods**: a data extraction form was used to capture data from the NHIF database. Specifically, the original yellow claim forms 2A & B which health-care facilities submit to NHIF headquarters were the data source. These NHIF claim forms included the health provider in/outpatient claim form and the health provider surgery claim form. Only the accessible claim forms or data sets were used as our data source, as we appreciated that it was difficult to access unprocessed forms.

**Data management and statistical analysis**: during and immediately after data collection, all data extraction forms were checked for completeness and omissions. Secondary reviews of the claim forms were warranted to ensure the correctness of data. Homogeneous subgroups were clustered together and then the data was coded to simplify the analysis. Coded data was then entered and analyzed in IBM SPSS Statistics Version 23 software. A summary of frequency and proportions of categorical variables was obtained using descriptive statistics whereas that of numerical variables was obtained from measures of central tendency and dispersion. Analyzed data was presented in text, tables, and charts.

**Ethical consideration**: the proposal for this study was reviewed and approved by the MUHAS Institutional Ethics Review Board (IRB). Permission to use the NHIF database was sought from the Director of NHIF and was granted. Data collection did not start until these approvals had been obtained. Confidentiality was maintained as no personal identifiers were collected by our data capture tool.

## Results

**Figure 1:**
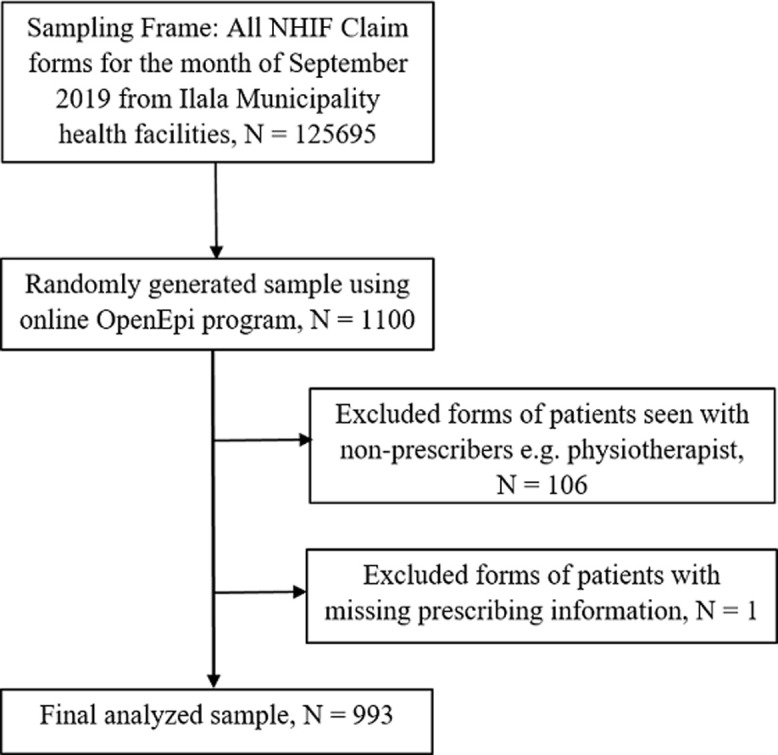
patient selection flow chart

A total of 1100 claim forms were reviewed and 107 were omitted from analysis due to either incomplete data or with non-prescriber status. Analysis was performed on 993 claim forms. The sampled claim forms were from a total of 73 NHIF-accredited health facilities of which 37 (50.7%) were dispensaries, 14 (19.2%) were health centers, 13 (17.8%) were National Referral Hospitals including level three clinics run by super-specialists, 3 (4.1%) were from Regional Hospitals including level two clinics run by specialists and 6 (8.2%) were from District Hospitals including level one clinics run by medical officers. The final analyzed sample is as shown in the flow chart (Figure 1). The majority of patients were females 581(58.5%) with mean (± Standard Deviation-SD) of 36.3 (±23.2) years ranging from 0 to 74 years. Majority of patients were seen at the outpatient department (98.2%) where they were mostly seen at private health care facilities (52.9%). Over half of the patients (55.2%) were attended at health facilities at the level of National Referral Hospitals. Moreover, the majority of the patients were seen by prescribers at the level of specialist (44.0%) (Table 1). Other select characteristics are also shown in Table 1.

**Table 1:** socio-demographic and other patient characteristics

Characteristic (N = 993)	n (%)
**Age in years**	
Mean (SD) = 36.3 (23.2), Median = 37.0	
Children (< 18 years)	264 (26.6)
Adults (18-59 years)	535 (53.9)
Elderly (≥ 60 years)	194 (19.5)
**Sex**	
Male	412 (41.5)
Female	581 (58.5)
**Level of health facility**	
Dispensary	102 (10.3)
Health Centre/Stand-alone clinic by Assistant Dental Officer	119 (12.0)
District Hospital/Clinic Level 1 by Medical/Dental Officer	101(10.2)
Regional Hospital/Clinic Level 2 by specialist)	123 (12.4)
National Referral Hospital/Zonal Hospital/Clinic Level 3 by super-specialist	548 (55.2)
**Ownership of health facility**	
Public	468 (47.1)
Private/Non-governmental	525 (52.9)
**Department visited**	
Outpatient	975 (98.2)
Inpatient	18 (1.8)
**Any Procedure/Surgery done**	
No	940 (94.7)
Yes	53(5.3)
**Prescriber Qualification**	
Clinical Officer/Dental Therapist	132 (13.3)
Assistant Medical/Dental Officer	18 (1.8)
Medical/Dental Officer	320 (32.2)
Specialist	437 (44.0)
Super-specialist/Consultant	86(8.7)

**Figure 2:**
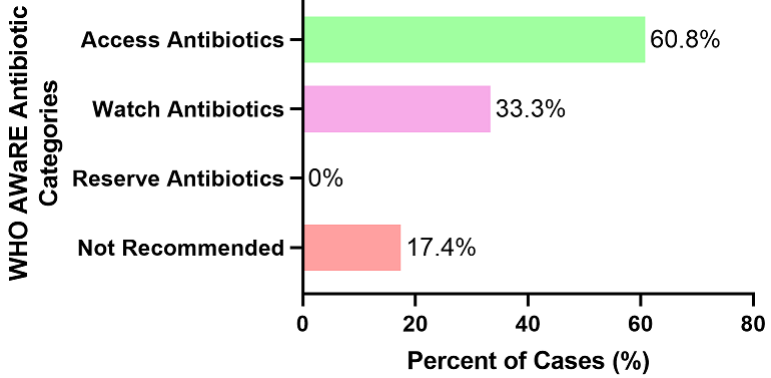
prescribed antibiotics as per 2019 WHO AWaRe classification

**Antibiotic prescription prevalence and pattern**: among patients who received a medication prescription, 46.4% (95% Confidence, CI; 42.8-50.0) contained one or more antibiotic medication. Furthermore, 92.2% of the antibiotics prescribed were in agreement with current Tanzania standard treatment guideline recommendation with respect to health facility level (2017 TZ STG/NEMLIT). The most common antibiotic name prescribed was amoxicillin/clavulanate (17.1%) followed by amoxicillin (16.5%) whereas the most commonly prescribed antibiotic class was the penicillins (51.3%) followed by the nitroimidazoles (14.0%) (Table 2). Nearly half (48.2%) of the antibiotics prescribed were in tablet formulation whereas the most commonly prescribed antibiotic frequency was twice daily (47.3%). The median duration of antibiotic treatment was five days ranging from 1 day to 30 days. A higher proportion of patients (60.8%) received the 2019 WHO AWaRe access group of antibiotics whereas 17.4% of patients received antibiotics which were in the WHO not recommended group (Figure 2). No patient received an antibiotic from the reserve group of drugs. The proportion of patients who received more than one antibiotic was 19.6%. Among patients who received more than one antibiotic, the most commonly co-prescribed antibiotics were Ampicillin/Cloxacillin plus Metronidazole (11.4%) followed by Amoxicillin plus Metronidazole (7.1%). The top ten co-prescribed antibiotics are as shown in Table 3.

**Table 2:** top ten antibiotic names and classes prescribed

Characteristic (N = 357)	n (%)
**Antibiotic Name prescribed**	
Amoxicillin/Clavulanate	61 (17.1)
Amoxicillin	59(16.5)
Ampicillin/Cloxacillin	53(14.8)
Metronidazole	38(10.6)
Ciprofloxacin	34 (9.5)
Ceftriaxone	20(5.6)
Azithromycin	18(5.0)
Erythromycin	18(5.0)
Trimethoprim/Sulfamethoxazole (cotrimoxazole)	18(5.0)
Gentamycin	13(3.6)
Mupirocin	13 (3.6)
Neomycin	13(3.6)
Cefixime	8(2.2)
Tinidazole	8 (2.2)
Cephalexin	7 (2.0)
Clarithromycin	7(2.0)
**Antibiotic Class prescribed**	
Penicillins	183 (51.3)
Nitroimidazoles	50 (14.0)
Macrolides	44(12.0)
Cephalosporins	38 (10.6)
Quinolones	36(10.1)
Aminoglycosides	26(7.3)
Sulfonamides	19 (5.3)
Pseudomonic acids	13 (3.6)
Tetracyclines	8(2.2)
Quinolone/Nitroimidazole Combos	6(1.7)

**Table 3:** the top ten co-prescribed antibiotics

Co-prescribed antibiotics (N = 70)	n (%)
Ampicillin/Cloxacillin+Metronidazole	8(11.4)
Amoxicillin+Metronidazole	5(7.1)
Ceftriaxone+Metronidazole	4(5.7)
Ceftriaxone+Gentamycin	3(4.3)
Amoxicillin/Clavulanate+Mupirocin	2(2.9)
Amoxicillin/Flucloxacillin+Gentamycin	2 (2.9)
Cephalexin+Metronidazole	2 (2.9)
Ciprofloxacin+Metronidazole	2(2.9)
Amoxicillin/Clavulanate+Metronidazole	2(2.9)
Amoxicillin+Tinidazole	2(2.9)

## Discussion

In this cross-sectional analysis of insured patients attending health facilities in an urban setting, we aimed to determine the prevalence and describe the pattern of antibiotic prescriptions. The prevalence of an antibiotic prescription was high (46.4%). The most common antibiotic name prescribed was amoxicillin/clavulanate whereas the most common antibiotic class prescribed was the penicillins. The most commonly co-prescribed antibiotics were ampicillin/cloxacillin plus metronidazole whereas, as per WHO AWaRe classification, the most commonly prescribed antibiotics were from the Access group.

We found that antibiotic prescriptions are highly prevalent (46.4%) with 19.6% of the patients receiving more than one antibiotic. This finding is above the ideal antibiotic prescription rate of 30.0%, therefore, indicating over prescription [9]. This suggests that nearly half of patients attending health facilities in the study area are exposed to antibiotics. This may have negative implications in the fight against antibiotic resistance. A similar observation was demonstrated by a study in Nigeria among insured patients which found an antibiotic prescription prevalence of 46.9% [19]. Systematic reviews for drug utilization patterns in the global context and that of prescribing indicators at Primary Health Care (PHC) within the WHO African Region revealed an antibiotic prescription prevalence of 50.0% and 46.8% respectively [20,21]. A previous study by Irunde and colleagues in Tanzania, revealed a higher antibiotic prescription prevalence of 67.7% [7]. However, this high prevalence could also be explained by their study population being largely from Primary Health Care whilst most of our study subjects were from tertiary care facilities. At tertiary level, lower antibiotic prescription prevalence could be attributed to the fact, there are more qualified prescribers who are more adherent to recent treatment guideline, which is more likely to be available at this level.

The observation that amoxicillin/clavulanate was the most commonly prescribed antibiotic, and penicillins were the most common antibiotic class is consistent with previous studies [19,22-25]. This could be explained by our study population being insured, therefore more likely to be prescribed an expensive and more profitable beta-lactamase penicillin, amoxicillin/clavulanate, rather than amoxicillin. The second most commonly prescribed antibiotic class was the nitroimidazoles. This observation was also consistent with a study done in Nigeria among insured patients [19]. Contrary to our finding, a recent article on National Consumption of Antimicrobials in Tanzania (2017-2019) showed that the most commonly consumed antimicrobial was doxycycline in the tetracycline class, however, it was based on importation, purchasing and local manufacturing data and not on prescription data [26].

Our data show that the Access group of antibiotics is the most commonly prescribed antibiotics among insured patients in Tanzania. However, the level at which these antibiotics are prescribed is still low (60.8%), just above the WHO country-level recommendation of 60.0% [12]. The finding that the Access group of antibiotic is the most commonly prescribed is consistent with recently published studies [26,27]. It should be noted that, our study involved only insured patients and the combined (insured and non-insured patients) Access antibiotic prescription level in Tanzania is unknown but it may be low since uninsured patients are less likely to receive an antibiotic prescription [13,14]. More effort is needed, where indicated, to increase the prescription of antibiotics from the Access group. Stewardship programs and monitoring should target the Watch group antibiotics so as to increase, where appropriate, the prescription of access antibiotic [12]. Moreover, a significant proportion of antibiotics which are not recommended as per 2019 WHO AWaRe Classification were also prescribed. These include fixed-dose combinations of broad spectrum antibiotics which lack evidence based indication [12]. This observation may be explained by presence of these antibiotics in the recommended standard treatment guideline and the National Essential Medicine List (NEMLIT) of 2017 [28].

This study has some limitations. Firstly, there is lack of generalizability of the study findings due to the involvement of one municipality and among insured patients only. However, this may reflect a similar or worse trend in other areas of the country and among uninsured patients. Secondly, we could not control for seasonal bias in antibiotic prescription prevalence and pattern as we used retrospective data over one-month period.

## Conclusion

This study has highlighted that, among insured patients, the prevalence of antibiotic prescriptions is high and antibiotics of fixed-dose combinations which are not recommended by the WHO are still prescribed. We recommend, the standard treatment guidelines currently in use should be revised to reflect the current WHO recommendation especially on removing the antibiotics which are not recommended and further research to address local factors that may influence an antibiotic prescription is warranted.

### What is known about this topic

- Antibiotics are over prescribed globally;
- Antibiotics over prescription may lead to drug resistance;
- Understanding the prevalence and pattern of antibiotic prescriptions may inform public health interventions in combating increased antibiotic use.

### What this study adds

- Antibiotic prescription among insured patients in Tanzania is high;
- Access antibiotics prescribed met the WHO country level recommendation;
- WHO un-recommended antibiotics are still prescribed among insured patients in Tanzania.
